# Mesenchymal stem cell-mediated delivery of therapeutic adenoviral vectors to prostate cancer

**DOI:** 10.1186/s13287-019-1268-z

**Published:** 2019-06-25

**Authors:** Tahir Muhammad, Ali Sakhawat, Aamir Ali Khan, Ling Ma, Ruth A. Gjerset, Yinghui Huang

**Affiliations:** 1 College of life sciences and Bio-engineering, Beijing University of Technology, Beijing, China; 2 Torrey Pines Institute for Molecular Studies, San Diego, CA USA

**Keywords:** Mesenchymal stem cells, Prostate cancer, Adenoviral vectors, Apoptosis, p53

## Abstract

**Background:**

There is an urgent need for targeted biological therapies for prostate cancer with greater efficacy and less toxicity, particularly for metastatic disease, where current therapies are not curative. Therapeutic adenoviral vectors or oncolytic adenoviruses offer the possibility of a competent, nontoxic therapeutic alternative for prostate cancer. However, free viral particles must be delivered locally, an approach that does not address metastatic disease, and they display poor tumor penetration. To fully exploit the potential of these vectors, we must develop methods that improve intratumoral dissemination and allow for systemic delivery. This study establishes a proof-of-principle rationale for a novel human mesenchymal stem (stromal) cell-based approach to improving vector delivery to tumors.

**Methods/results:**

We have generated mesenchymal stem cell-derived packaging cells for adenoviruses (E1-modified mesenchymal stem cells) by modifying human mesenchymal stem cells with the adenovirus (type C) E1A/B genes needed for viral replication. Using cell-based assays, we have demonstrated that two adenoviral vectors, replication-defective adenovirus expressing p14 and p53 or conditionally replicating oncolytic adenovirus, packaged by E1A/B-modified mesenchymal stem cells, suppress the growth of prostate cancer cells in culture. Using subcutaneous xenograft models for human prostate cancer in mice, we have shown that E1A/B-modified mesenchymal stem cells display tumor tropism in tumor-bearing nude mice, that E1A/B-modified mesenchymal stem cells disseminate well within tumors, and that replication-defective adenovirus expressing p14 and p53 or conditionally replicating oncolytic adenovirus-loaded E1-modified mesenchymal stem cells suppresses tumor growth in mice.

**Conclusion:**

The results show that this approach, if optimized, could circumvent the obstacles to efficient gene delivery encountered with current gene delivery approaches and provide an effective, nontoxic therapeutic alternative for metastatic disease.

## Introduction

Prostate tumors are the second leading tumors in men globally [1, 2], with about 1.6 million new cases registered in 2015 [3]. The global problem of prostate cancer is significant, in terms of occurrence and cancer-related death rate. It is included in the top five cancers [4]. Well-known risk factors for overall prostate cancer occurrence include older age, family history of prostate cancer, and African American ethnicity. Currently, genome-wide association studies on prostate cancer revealed the role of genetic predisposition in the onset of prostate cancer [5, 6]. Chemotherapy, radiotherapy, androgen deprivation therapy (ADT), active surveillance (AS)/watchful waiting (WW), and surgery are the current treatment strategies for prostate cancer. However, the most suitable choice of treatment strategy is still unclear [7]. Therefore, less toxic, more efficient, and targeted biological therapies for prostate cancer are urgently needed to develop, mainly for metastatic prostate cancer, where currently practicing therapies are not much useful. In the past few years, researchers are working on developing and improving the approaches to preferentially deliver the genes of choice (therapeutic genes) to tumor site as well as selective expression of therapeutic genes specifically on tumor sites. Both considered as important mechanisms for successful gene therapy [8]. Current innovations in genetic engineering techniques and gene therapeutics have urged the need to use viral-based vectors for therapeutic genes delivery [9]. Adenoviral vectors were the first viral-based vectors used in humans for gene therapy, considered to be an attractive option due to harboring particular merits like high transduction efficiency, comparatively low toxicity and cost-effective production at a large scale [10]. The possibility of adenovirus-based vectors to be used in humans for gene therapy is proved by the approval of the first gene therapy agent, an adeno-associated virus (AAV) in the USA and Europe [11]. For systemic delivery of therapeutic genes to tumor sites, a variety of viral, synthetic, and cell-based vectors have engineered, but most have shown low practical efficiency [12]. High doses of viral vectors (> 10^12^ particles) were required to inject directly into tumors to achieve satisfactory antitumor effects. Intravenous injections of viral vectors were also proved less efficient, as most virus particles fail to disseminate into the tumors and eventually sequestered by the reticuloendothelial system [13, 14]. Moreover, gene-based and virus-based therapies have exhibited limited achievement due to the activation of the host immune system in response to the viral vectors [15, 16]. The development of new approaches is urgently required to transfer therapeutic genes selectively to tumor sites, to disseminate the therapeutic vectors specifically into the tumors and to address the metastatic condition of disease while avoiding the adverse effects to healthy cells. Mesenchymal stem cells are attractive vehicles for gene therapy due to their outstanding ability to migrate towards tumor lesions and homing at tumor site [17]. Moreover, MSCs can preferentially and accurately target tumor lesions after intravenous [18], intra-arterial [19, 20], and intracardiac injections [21, 22].

In different studies conducted to examine the effectiveness of adenoviral-based therapies, mainly two different types of adenoviral vectors are employed. One type is conditionally replicating adenoviral vectors, which tend to propagate in and lyse cancer cells. The other is replication-defective adenoviral vectors, which cannot replicate by their own but contain therapeutic genes and deliver to tumor cells, and once reaching there, these genes expressed and killed the tumor cells according to their specific mode of action. In our study, we have used both types of adenoviral vectors like conditionally replicating adenoviruses under the control of survivin promoter (CRAds) which specifically and selectively replicate in the tumor cells and cause oncolysis and replication-defective adenoviruses containing p14ARF/p53 tumor suppressor genes (Adbic). These both adenoviral vectors have shown the effective and nontoxic antitumor activity. To obtain optimum delivery and maximum antitumor effects of these adenoviral vectors, we have developed a novel vehicle system for adenoviral vectors delivery by equipping human mesenchymal stem (stromal) cells (MSCs) with adenovirus (type C) E1A/B (E1s) genes. These genes are vital for the replication of adenoviruses. In our study, we have constructed novel MSCs-E1s for propagation and targeted delivery of therapeutic adenoviral vectors to prostate tumor. Findings of this study support that MSCs-E1-mediated delivery of adenoviral vectors not only overcame the hurdles to systemic delivery of free adenoviral vectors but also resulted in improved intratumoral dissemination of these vectors. That makes it an effective and nontoxic approach to get maximum antitumor effects of adenoviral vectors against prostate cancer.

## Material and methods

### Cell lines

Prostate cancer cell lines LNCaP (ATCC, CRL 1740) and C 4 – 2 (ATCC, CRL 3314) procured from ATCC. Primary human stem cells (MSCs) were purchased from Bio Whittaker/Cambrex Bio Science, USA. Immortalized human stem cells Hs27a were kindly provided by Beverly Torok-Storb, and these were maintained and grown as previously described [23]. Dr. Johng Rhim kindly provided Ki-ras 267B1 cells, and these were also maintained and grown as previously described [24].

### Adenoviral vectors

Conditionally replicative oncolytic adenoviruses (CRAds) were prepared by placing the E1A region of adenovirus under the control of survivin promoter and by deletion of E1B region of adenoviruses. Furthermore, a reporter gene luciferase was introduced in these adenoviruses. Survivin promoter tends to express selectively in tumor cells with absent or low expression in normal cells. Replication-defective adenovirus expressing p14 and p53 (Adbic), reported by Huang et al. [25], were kindly provided by Dr. Yinghui Hunag.

### Subcutaneous tumor model

Six- to 8-week-old male BABL/C nude mice were procured. These mice were injected subcutaneously with 0.1 ml of Ki-ras transfected cells (3 × 10^6^ cells/ml) into the hind flank. Tumors appeared after 2 weeks of Ki-ras cell inoculation. In vivo experimentations using mice tumor models were carried out by following ARRIVE guidelines [26].

### Spheroids tumor model

Prostate tumor cells spheroids were prepared by pouring 100ul of TRAMP C2 H2B/GFP prostate tumor cells (2.5 × 106 cells/ml) into each well of 96-well plates in DMEM containing 10% FBS. Wells were coated with 2% agarose. Spheroids were allowed to solidify for 48 h at 37 C in 5% CO2. Compacted spheroids were collected and washed with PBS. Then, these spheroids were implanted in the dorsal skinfold chambers in nude mice as previously described [27–29]. An epi-illuminator-attached fluorescence microscope (Mikron Instruments, San Diego, CA, USA) was used to execute the intravital microscopy. A silicon-intensified target camera (SIT68; Dage-MTI, Michigan City, IN) attached to the microscope was used for fluorescence imaging. Tumor growth in the dorsal skinfold chamber was visualized by using a GFP C 3902 filter cube (Chroma, Battleboro, VT). Cell viability was ensured by visualizing the intrinsic fluorescence of H2B–GFP and chromatin integrity monitored under high-power field microscopy (× 20 and × 63). Five days following implantation of spheroids, chambers were injected with respective adenoviral vectors and observed accordingly. In vivo experimentations in mice tumor models carried out by following ARRIVE guidelines [26].

### Cell viability assay

Prostate cancer cells were cultured in 24-well plates and treated with CRAd and Adbic adenoviral vectors in DMEM containing 2% FBS for 4 h at different multiplicity of infection (MOI), then cells were shifted to 96-well plates (three wells for each treatment group and control group) with a concentration of 3000 cells/well in complete medium. After 48 h, 20 μl of MTT (5 mg/ml) reagent was added to each well and incubated for 4 h, and then the medium was removed from the wells, and 200 μl of DMSO was added to each well and incubated for 10 min. After incubation, the OD of each well was taken at 490 nm by using ELISA microplate reader. Viability was expressed as a percentage of uninfected cell viability.

### Apoptosis analysis

Prostate cancer cells C4–2 and LNCaP were cultured in 6-well plates, and tumor cells (1 × 10^6^ cells/well) were treated separately with both CRAd and Adbic adenoviral vectors at different MOI. After 48 h, cells were collected and apoptosis analysis was performed by using the Annexin V-FITC/Propidium iodide (PI) Apoptosis Detection kit (Invitrogen, Carlsbad, CA, USA) according to the procedure provided by the manufacturer. Apoptosis was measured by using FACScalibur™ (Becton Dickinson) flow cytometer.

### Western blotting analysis

Prostate cancer cells were cultured and treated with CRAd and Adbic adenoviral vectors at different MOI. After 48 h, cells were collected and treated with RIPA lysis buffer for 30 min on ice to obtain cell lysate. Thirty micrograms of total protein was electrophoresed on 15% polyacrylamide gel electrophoresis and transferred onto PVDF membranes (Millipore, Billerica, MA, USA). Membranes were then subjected to treatment with primary antibodies against p53 (1:500), Mdm2 (1:500), p21 (1:500), Bax (1:500), Bcl2 (1:500), and β-actin (BIOSS Antibodies, Boston, USA). Membranes were incubated at 4 °C, overnight, then membranes were washed three times (15 min each) with TBS. After washing, the membranes were treated with secondary antibodies for 2 h at room temperature, then membranes were washed with TBS, and quantification of bands was performed by using Kodak digital camera and analysis software (Kodak, Rochester, NY, USA). The data were normalized to β-actin for analyses and plotting.

### Statistical analysis

The data sets were obtained after performing three independent experiments, and results were shown as the mean ± standard deviation. GraphPad Prism 5 software was used to perform statistical analysis. Data sets compared by using Student’s *t* test. *P* value < 0.05 was considered significant.

### Ethics statement

In this study, in vivo experiments involving mice conducted by following the ARRIVE guidelines for reporting experiments involving animals [26]. All animals were given proper care while carrying experimentations. In this study, the experimental animals were maintained on a twelve-hour light/dark cycle and kept in a temperature-controlled room at 22–24°C. The animals provided with free access to food and water. All experimental procedures conducted in this study approved by the internal ethical committee of the Faculty of Life Sciences and Biomedical engineering of the Beijing University of Technology (Number IRB-1507). At the end of treatment mice were euthanized by cervical dislocation. Manually performed cervical dislocation resulted in euthanasia within 10 – 15 seconds.

## Results

### Antitumor activity of CRAd and Adbic adenoviral vectors in vitro

**Fig. 1:**
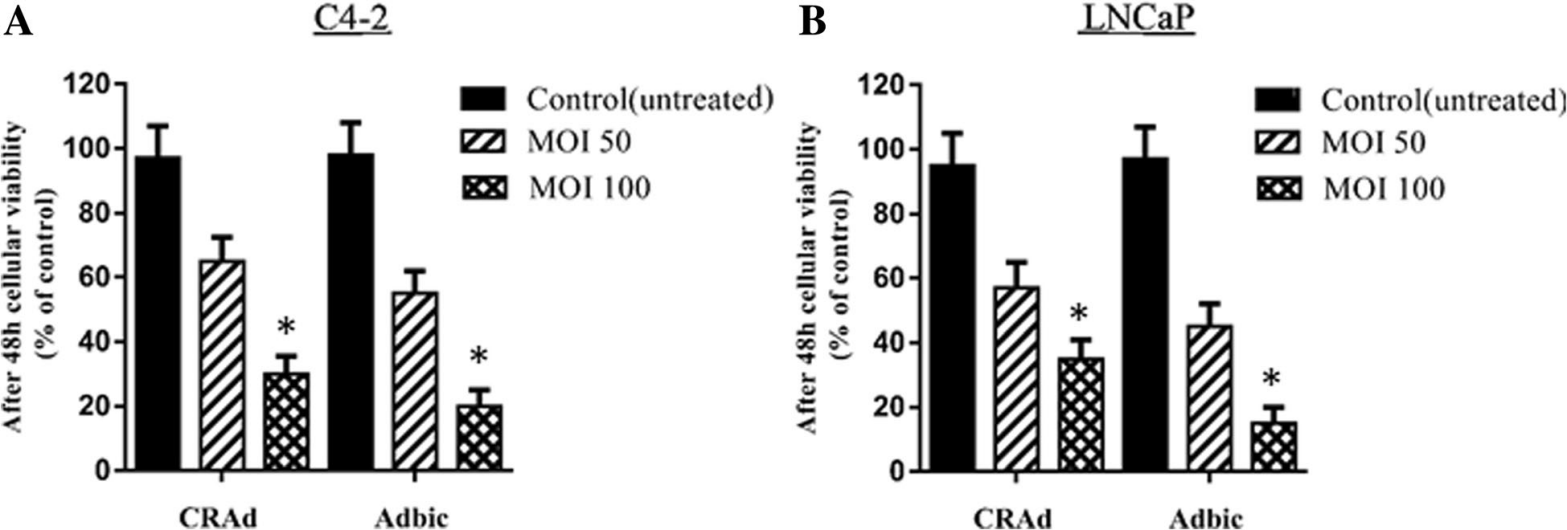
Percentage viability assay: **a**, **b** Viability of cells was measured by MTT assay after 48 h treatment of C4–2 and LNCaP cells with adenoviral vectors at 50 and 100 MOI. Histograms show that compared to control, the viability of treated cells significantly decreased at 100 MOI. Data in histograms represent the average of triplicate samples, with standard deviations shown (*P < 0.05)

We evaluated the in vitro antitumor activity of adenoviral vectors, 48 h after the treatment of C4–2 and LNCaP cells with CRAd and Adbic adenoviral vectors at 50 and 100 MOI. Both adenoviral vectors exhibited remarkable antitumor activity at 100 MOI concentration against prostate tumor cells as compared to control (untreated) cells as detected by MTT assay (Fig. 1).

### Apoptosis induction by adenoviral vectors in prostate tumor cells

**Fig. 2:**
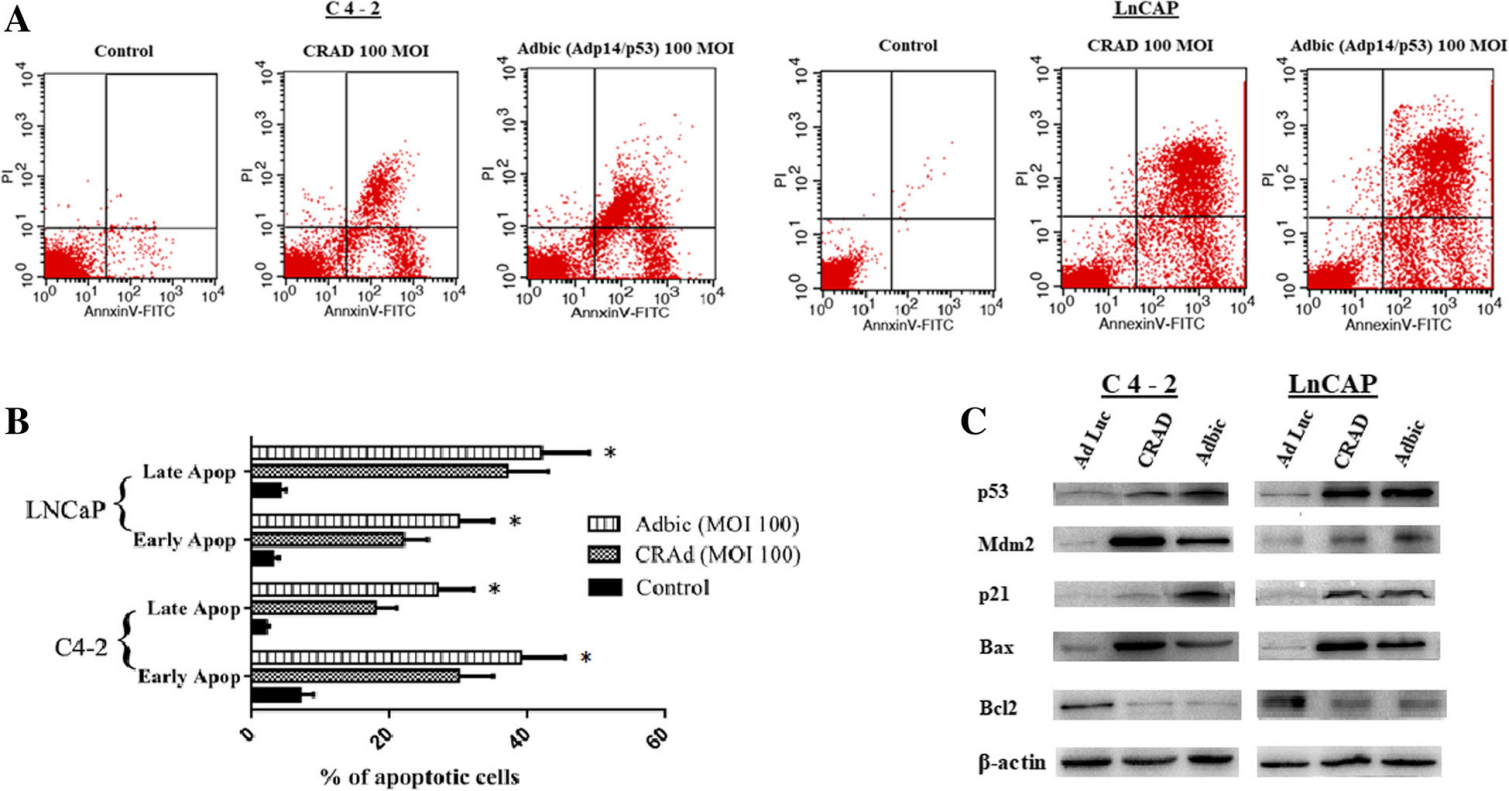
Apoptosis induced by CRAd and Adbic in prostate tumor cells. **a** Flow cytometry analysis by FASCcalibur. Prostate tumor cells stained with Annexin V-FITC/Propidium iodide stains after 48 h of treatment with CRAd and Adbic adenoviral vectors at 100 MOI. **b** Histogram showing the representative apoptosis rate in prostate tumor cells. Results showed that both adenoviral vectors significantly raised the apoptosis rate in both prostate cancer cell lines at 100 MOI. Data represent the average of the triplicate experiment (*P < 0.05). **c** Expression analysis of p53, Mdm2, p21, Bax, Bcl2 proteins in C4–2, and LNCaP cells lysate, performed 48 h after treatment of C4–2 and LNCaP cells with CRAd and Adbic. Thirty microgram of total protein lysate electrophoresed on each lane. Equivalent loading was verified by stripping membranes and re-probing with actin antibody

AnnexinV-FITC/ PI staining was used to measure the apoptosis induction in prostate tumor cells after treatment with adenoviral vectors at 100 MOI for 48 h. Results have shown that adenoviral vectors significantly induced the apoptosis in tumor cells as compared to control (untreated) cells. We observed that the rate of apoptosis was more in cells treated with Adbic as compared to cells treated with CRAd (Fig. 2a, b).

### Expression analysis of proteins associated with apoptosis

We performed western blot analysis on protein extract taken from C4–2 and LNCaP tumor cell lysate after treatment with adenoviral vectors for 48 h. We evaluated the expression of p53, Mdm2, p21, Bax, and Bcl2. We found that the expression of p53 protein was increased in both treatment groups as compared to control group. The expression of Mdm2 and p53 downstream genes p21 and Bax was also found out to be raised while the expression of Bcl2 was decreased in both the treatment groups as compared to the control group. We observed that the expression of Bax increased, and the expression of Bcl2 decreased in the treatment groups which are a strongly indicated apoptosis induction (Fig. 2c).

### Tumor suppression by free replication-defective adenoviral vectors

**Fig. 3:**
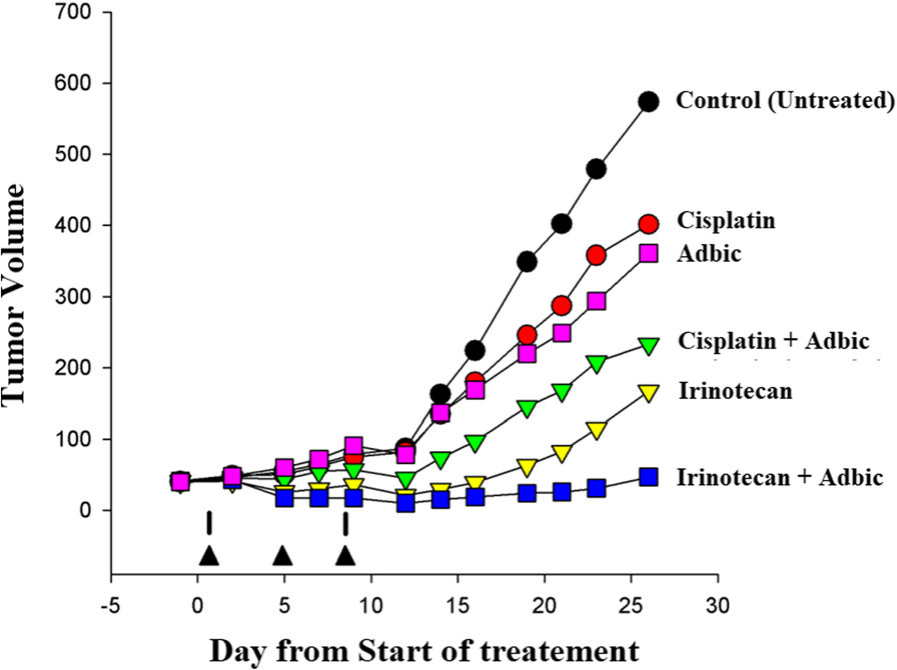
Free Adbic suppresses and chemosensitizes prostate tumors. Six treatment groups of male nude mice, each group containing seven mice, were treated differently with anti-cancer therapeutic agents. Free Adbic (3 × 10^7^ pfu/mouse) given on days 1, 5, and 9 of treatment and cisplatin (5 mg/kg) or irinotecan (40 mg/kg) given on days 1 and 9 of treatment as indicated by the arrows (▲). All the treatment groups, that either received monotherapy or combined therapy, showed reduced tumor growth as compared to the untreated (control) group

Forty-two male nude mice bearing subcutaneous Ki-ras cell tumors (vol ~ 50 mm^3^) were randomized into six groups of seven mice in each group, and different treatments were given to different mice model groups. At the end of treatment, the mice were euthanized, and tumor volumes were measured and compared. We administered one group of prostate tumor models with cisplatin (5 mg/kg) as monotherapy on the days 1 and 9 of treatment. Another group of prostate tumor models received Adbic (3 × 10^7^ pfu/mouse) as monotherapy on days 1, 5, and 9 of treatment. In the combined therapy group, we administered cisplatin (5 mg/kg) and Adbic (3 × 10^7^ pfu/mouse) together. Cisplatin was administered on days 1 and 9 of treatment, and Adbic administered on days 1, 5, and 9 of treatment. Mice model groups treated with cisplatin (monotherapy), Adbic (monotherapy), and cisplatin-Adbic (combined therapy) showed a reduction in tumor volume by 33% (vol ~ 400 mm^3^), 42% (vol ~ 350 mm^3^), and 62% (vol ~ 230 mm^3^), respectively, as compared to the control (untreated) mice group (tumor vol ~ 600 mm^3^). Similarly, mice groups received irinotecan (40 mg/kg) as monotherapy on days 1 and 9 of treatment and the combined therapy with irinotecan-Adbic (irinotecan on days 1 and 9 of treatment, and Adbic on days 1, 5, and 9 of treatment), which showed remarkable reduction in tumor volume by 70% (vol ~ 180 mm^3^) and 90% (vol ~ 60–70 mm^3^), respectively, as compared to the control (untreated) group (tumor vol ~ 600 mm^3^). Results showed that treatment groups that either received monotherapy with free replication-defective adenoviral vectors or chemotherapeutic drugs and combined therapy with both anti-cancer agents showed promising antitumor activity (Fig. 3).

### Intratumoral dissemination of free replication-defective adenoviral vectors

**Fig. 4:**
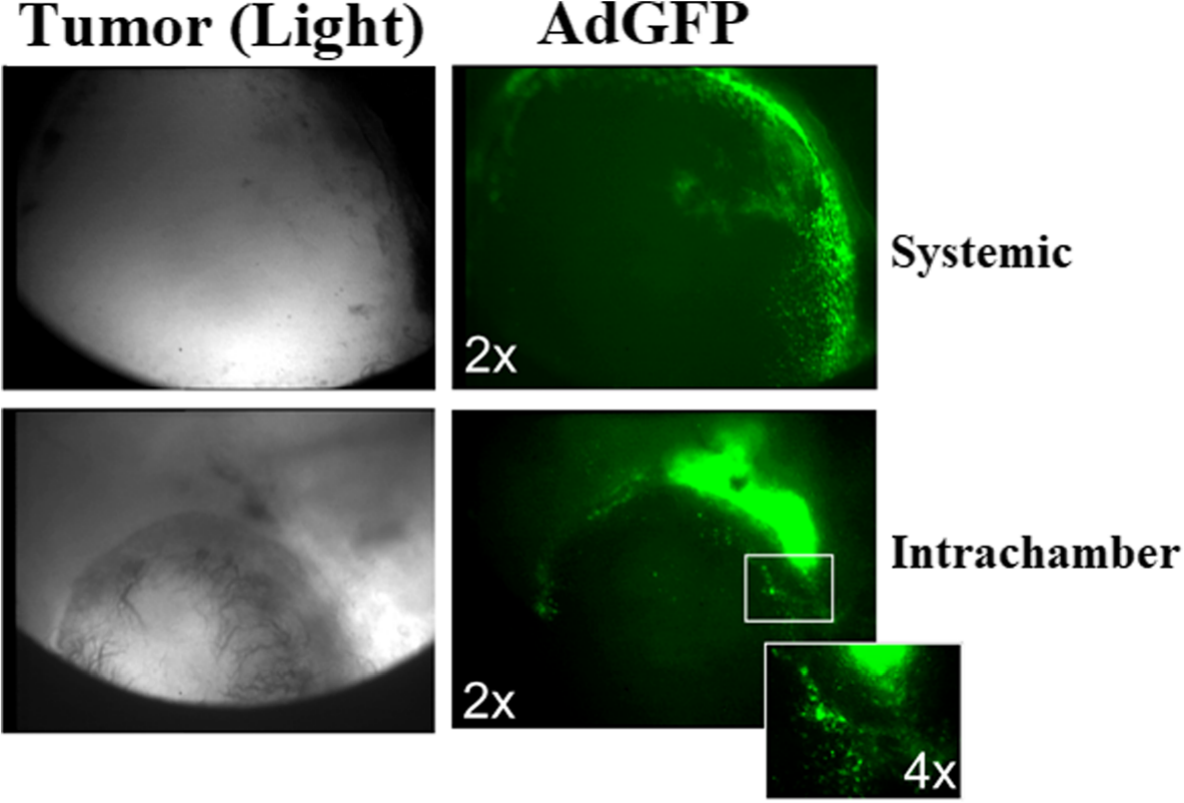
Free replication-defective adenoviral vector displays poor intratumoral dissemination. Mice models containing mouse TRAMPC2 prostate tumor spheroids, growing in dorsal skinfold chambers injected with replication-defective AdGFP. Four days later, intravital microscopy showed that free adenoviral vector chemo-sensitized prostate tumors but displayed poor intratumoral dissemination. AdGFP (administered via a tail vein or locally) remains mostly on the surface of the tumor

We also evaluated the tumor tropism and intratumoral dissemination of free replication-defective adenoviral vectors. For this purpose, we used nude mice implanted with TRAMPC2 prostate tumor spheroids in the dorsal skinfold chamber. These mice models were injected separately with replication-defective adenoviruses containing GFP (AdGFP) via tail vein (systematically) or locally. Chemosensitization to tumors and intratumoral dissemination of AdGFP was examined at day 4 post-AdGFP injection through intravital microscopy. Light and fluorescence images (× 2 magnification) of tumors in mice models receiving AdGFP systemically revealed that AdGFP successfully reached the tumor site, but their intratumoral dissemination was not much encouraging because these stayed mainly on the surface of tumors thus displaying poor intratumoral distribution. Tumor models that received AdGFP injection locally also exhibited a somewhat similar pattern of intratumoral dissemination. Light and fluorescence images (× 2, × 4 magnification) of tumors showed that AdGFP mainly remained at the injection site but did not disseminated well into the tumors (Fig. 4).

### Vector packaging by mesenchymal stem cells containing E1A/B genes

**Fig. 5:**
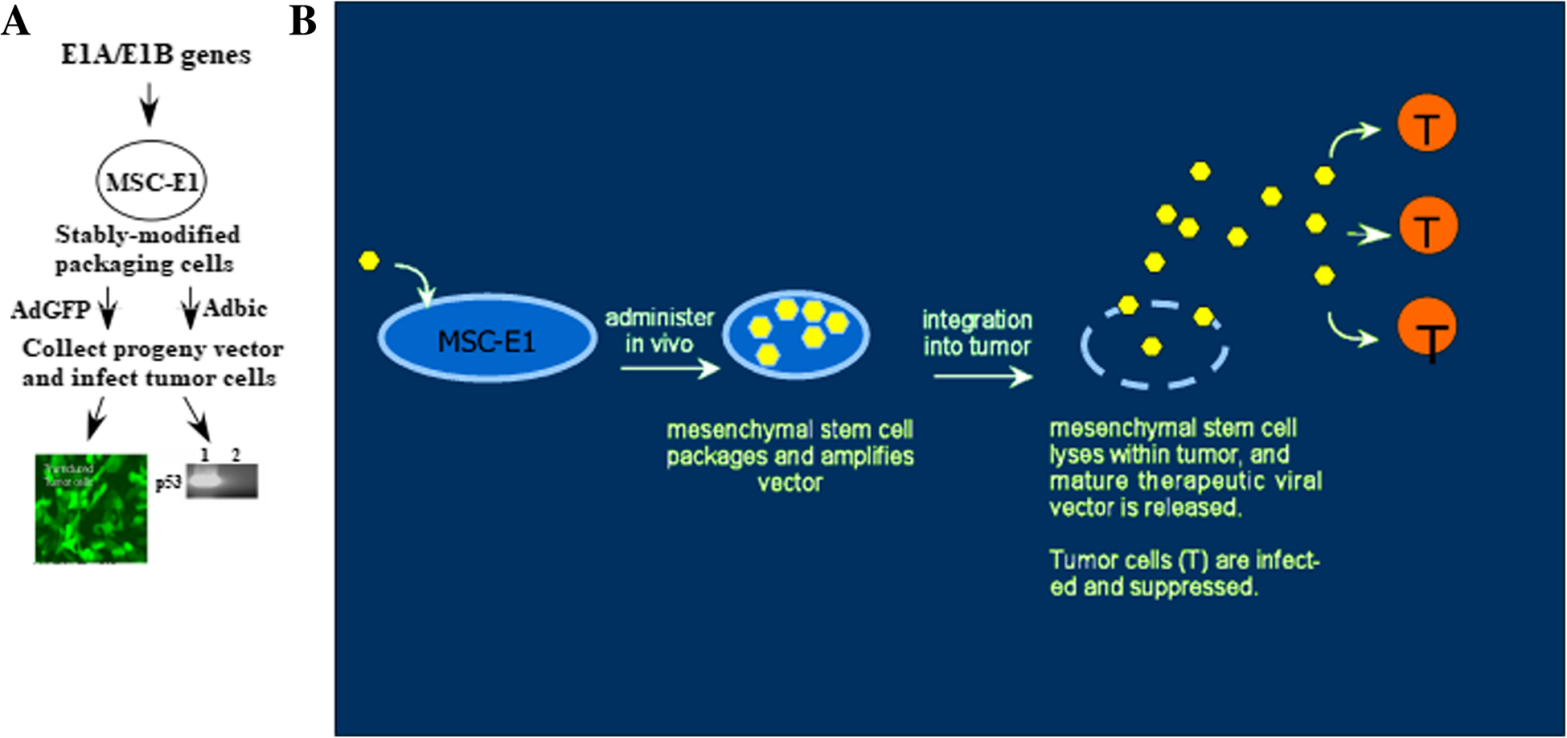
Packaging strategy of MSCs-E1s (**a**). MSCs-E1s successfully packaged and propagated replication-defective adenoviral vectors. We modified primary mesenchymal stem cells with E1A/B adenoviral packaging functions, MSC-E1s infected with replication-defective AdGFP and Adbic. MSCs-E1s loaded with AdGFP and Adbic successfully expressed GFP and p53 protein, respectively, in target tumor cells. **b** MODEL. MSCs-E1s are infected in a culture dish (ex vivo) with the viral vectors. Transfected MSCs-E1s are then administered in vivo, during which time they amplified the vector. Following integration into the tumor, the amplified vector released, where it can infect tumor cells (T) and suppress tumor cell growth

To obtain maximum anti-cancer therapeutic effects of adenoviral vectors, especially against metastatic cancer, we proposed a vehicle system for adenoviral vectors which is suitable for systemic delivery as well as will allow maximum intra-tumoral dissemination of adenoviral vectors. Here, we offer a model to address these issues (Fig. 5b).

Primary mesenchymal stem (MSCs) were transfected with E1A/B gene constructs to obtain MSCs-E1s. The construction of E1A/B gene plasmids were carried out as previously described [30]. Co-transduction of primary MSCs with these E1A/B gene constructs was performed by using DreamFect™ Stem transfection kit (OZ Biosciences INC, CA, USA) and following the protocol provided by the manufacturer. These modified mesenchymal stem cells were infected with replication-defective adenoviral vectors AdGFP and Adbic with different MOI. When the cytopathic effects appeared, progeny vectors collected from MSCs-E1 supernatant and tumor cells were infected, and after 48 h, the presence of GFP and p53 expression in tumor cells confirmed that adenoviral vectors AdGFP and Adbic were successfully propagated in MSCs-E1 packaging cells (Fig. 5a). The packaging and propagation efficiency of the MSCs-E1s were tested by loading these cells with adenoviral vectors and subjected to prostate tumor cells in vitro and in vivo.

### Tumor tropism and intratumoral dissemination of MSCs-E1s

**Fig. 6:**
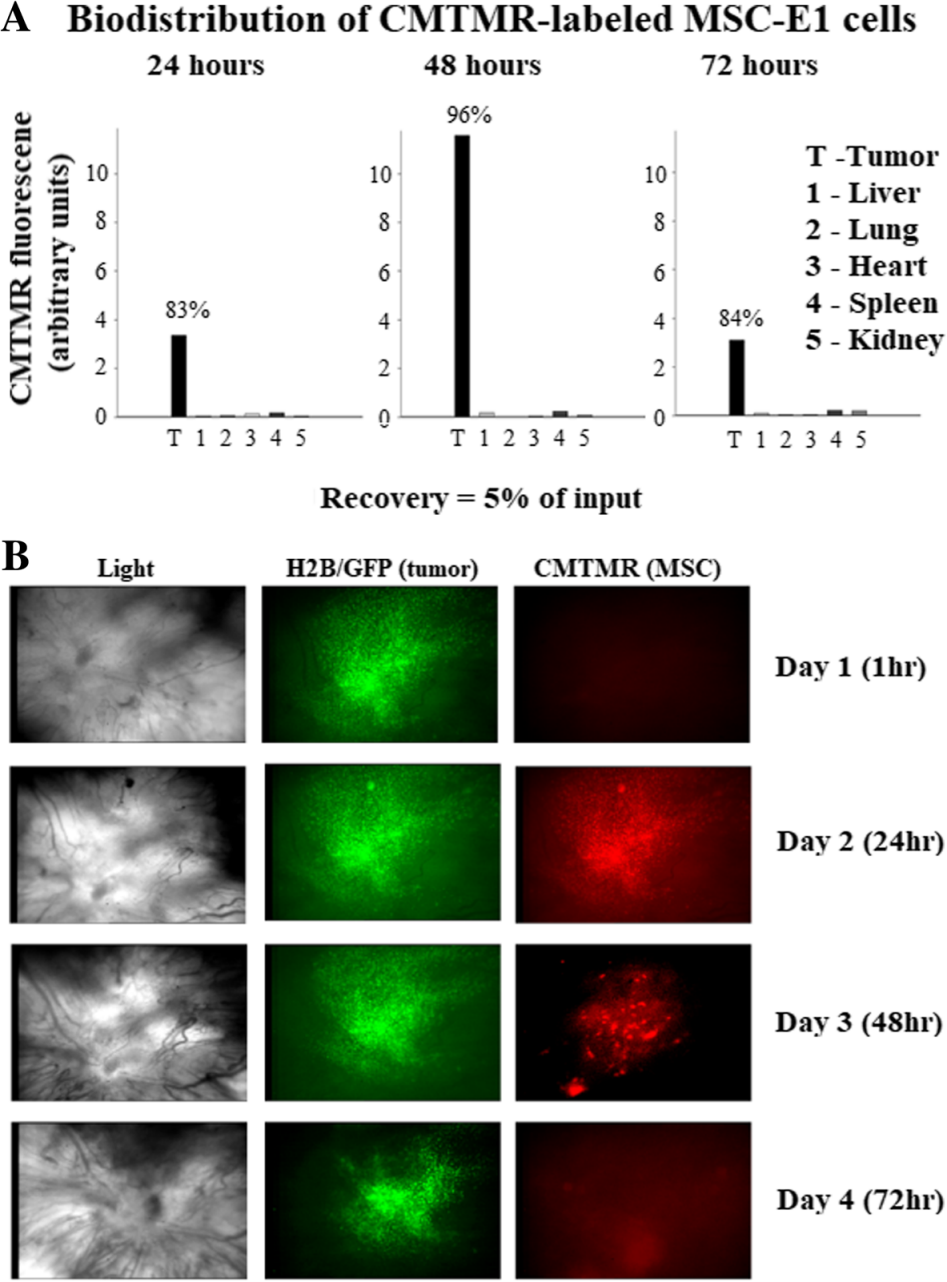
MSCs-E1s display tumor tropism and good intratumoral dissemination. **a** MSCs-E1s displayed promising tumor tropism. Nude mice bearing subcutaneous Ki-ras prostate tumors received CMTMR-labeled human MSCs-E1s (intracardiac administration). Mice were euthanized at 24, 48, and 72 h post-injection; tumors and tissues were excised, homogenized in RIPA buffer on ice, and fluorescence was measured. Bars indicate % of total recovery of fluorescence. **b** MSCs-E1s display good intratumoral dissemination. Intravital microscopy (× 4) showing TrampC2 tumor spheroids (Green) growing in dorsal skinfold chambers in nude mice. 3 × 10^6^ CMTMR-labeled MSCs-E1s (red) given via intracardiac administration. Observations were made at 1 h (top row), 24 h (second row), 48 h (third row), and 72 h (fourth row). The first column shows light images of chambers. Middle column shows green fluorescence of tumor. The last column shows red fluorescence of MSCs-E1s that are well disseminated within tumor masses

Nude mice bearing subcutaneous Ki-ras prostate tumors were injected with 3 × 10^6^ CMTMR-labeled MSCs-E1s. Mice euthanized at 24, 48, and 72 h and their organ tissues were surgically removed and homogenized in RIPA buffer on ice, and bio-distribution of MSCs-E1s was checked by measuring fluorescence. Fluorescence recovery was weak from normal vital organs, but fluorescence recovery was high from tumors (Fig. 6a). Intravital microscopy (× 4) of TRAMPC2 H2B/GFP tumor spheroids growing in nude mice was performed to evaluate the intratumoral dissemination of MSC-E1s followed by intracardiac administration of 3 × 10^6^ CMTMR-labeled MSCs-E1s to mice models. Microscopic observations at 1 h and daily intervals for 3 days showed that MSCs-E1s disseminated well intratumorally (Fig. 6b).

### Antitumor activity of vector-loaded MSCs-E1s in vitro

**Fig. 7:**
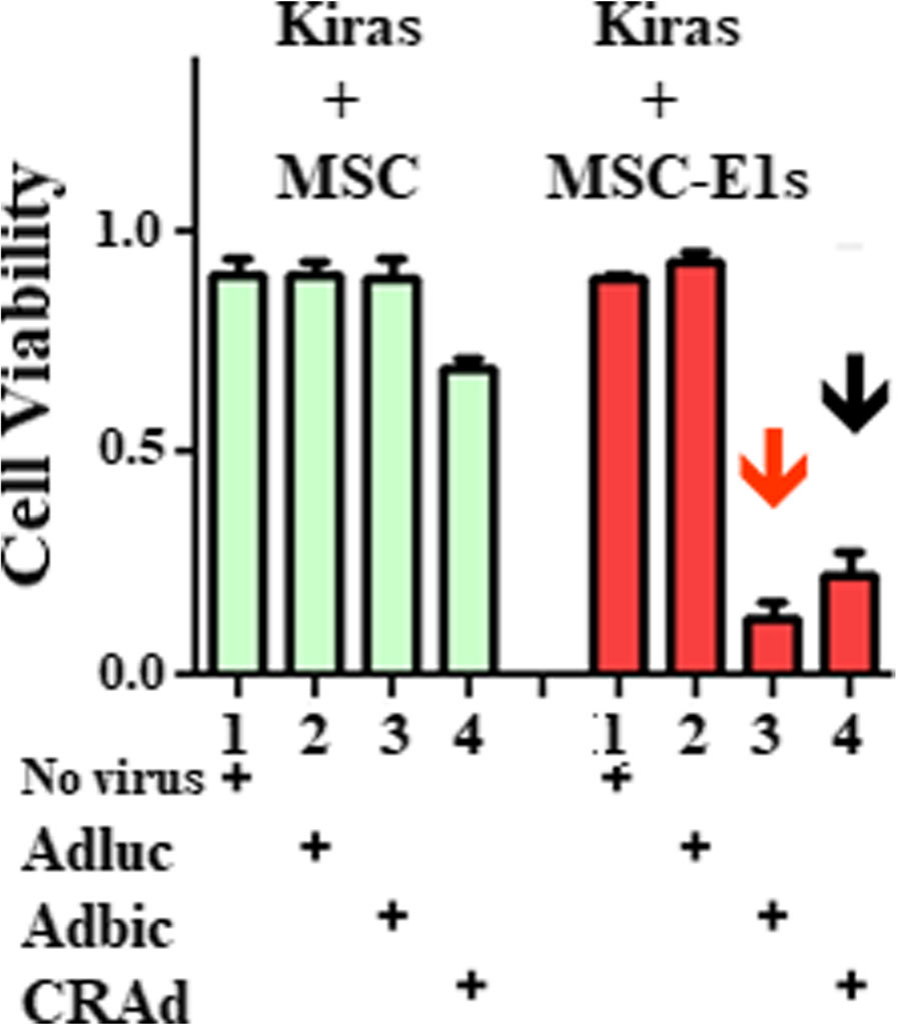
Adbic-loaded and CRAd-loaded MSCs-E1s suppress prostate cancer cells. Co-cultivation of Ki-ras cells with CRAd-loaded MSCs or MSCs-E1s and Adbic-loaded MSCs or MSCs-E1s was done. Five days later, MTS assay was performed, which showed that MSCs-E1s modification improved CRAd production () as well as enabled amplification of replication-defective Adbic () as compared to unmodified MSCs

Immortalized MSCs (Hs27a) were transiently modified with E1A/B. MSCs-E1s and unmodified MSCs were loaded with replication-defective adenoviral vector Adbic and CRAds. Once cytopathic effects were observed, cells were subjected to freeze/thaw and Ki-ras prostate cancer cells added. Five days after infection, MTS assay showed that MSCs-E1s exponentially propagated the viral vectors as compared to unmodified MSCs and in turn, viral vectors exhibited the noteworthy antitumor activity, in vitro (Fig. 7).

### Tumor suppression by adenoviral vector-loaded MSCs-E1s

**Fig. 8:**
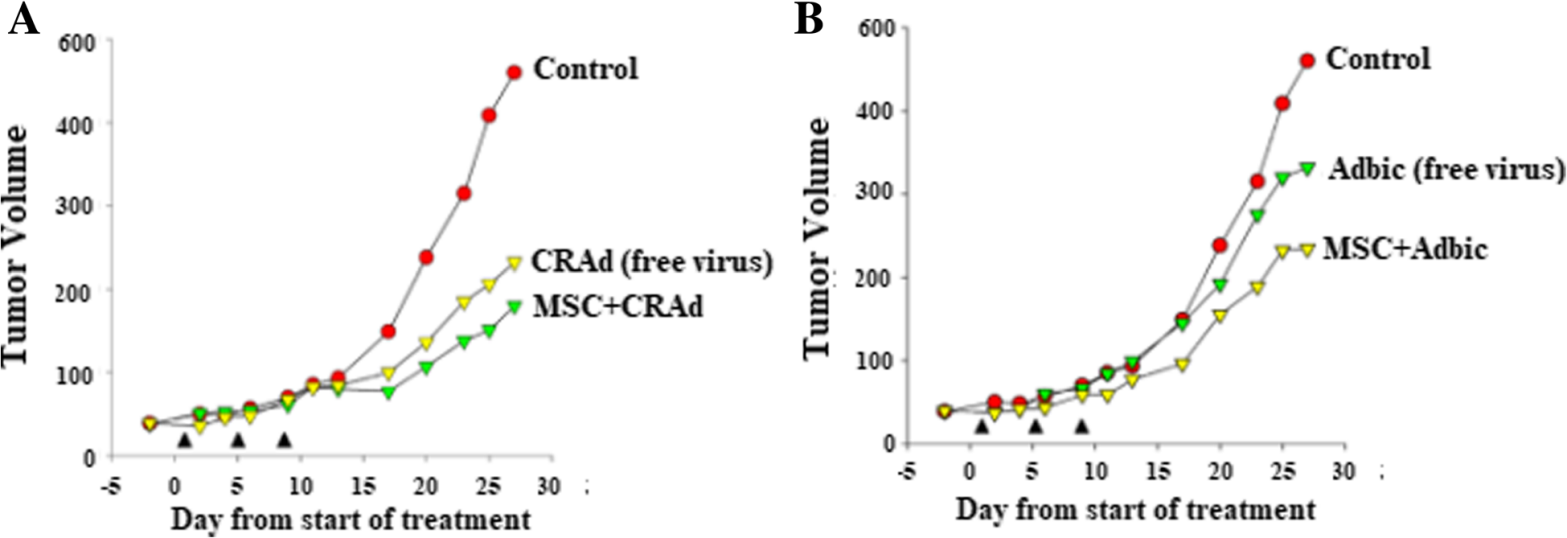
CRAd-loaded MSCs-E1s or Adbic-loaded MSCs-E1s have antitumor effects on subcutaneous human Ki-ras prostate tumors in nude mice. **a**, **b** Male nude mice bearing subcutaneous Ki-ras cell tumors (vol ~ 50 mm^3^) were randomized into five groups of seven mice and intratumoral treatments were initiated (day 1). Experimental groups for CRAd received either 5 × 10^5^ MSCs-E1s loaded with 3 × 10^7^ pfu/cell of CRAd vector or free CRAd vector (3 × 10^7^ pfu/mouse). Experimental groups for Adbic received either 5 × 10^5^ MSCs-E1s loaded with 3 × 10^7^ pfu/cell of Adbic or free Adbic (3 × 10^7^ pfu/mouse). Treatments [see arrows (▲)] were repeated on days 5 and 9 of treatment. Control groups did not receive any treatment. At the end of the treatment, mice models were euthanized and tumor volumes measured by the formula (tumor volume = ½ *L* × *w*^2^). Results showed that both types of treatments have significantly (*P* < 0.05) reduced the tumor volume as compared to the control group

Thirty-five male nude mice bearing subcutaneous Ki-ras cell tumors (vol ~ 50 mm^3^) were randomized into five groups of seven. Mice model groups received different treatments with free CRAds or Adbic and CRAds, or Adbic-loaded MSCs-E1s. Two treatment groups received either free CRAds (3 × 10^7^ pfu/mouse) or 5 × 10^5^ MSCs-E1s loaded with CRAds 3 × 10^7^ pfu/cell, and the other two treatment groups received either free Adbic (3 × 10^7^ pfu/mouse) or 5 × 10^5^ MSCs-E1s loaded with Adbic 3 × 10^7^ pfu/cell. The control group did not receive any treatment. Treatments repeated on days 5 and 9 of treatment as indicated by the arrows ▲ (Fig. 8a, b). At the end of treatment, mice were euthanized, and tumors from all treatment groups and the control group were excised and compared. Tumor volume was measured by the formula Tumor volume = ½ *L* × w^2^. Tumor volume was notably reduced in all the treatments groups as compared to the control group. Treatment groups received free CRAds, and CRAd-loaded MSCs-E1s showed 50% (average vol ~ 240 mm^3^) and 63% (average vol ~ 180 mm^3^) tumor reduction as compared to control group (average vol ~ 480 mm^3^), Similarly treatment groups received free Adbic, and Adbic-loaded MSCs-E1s exhibited 34% (average vol ~ 320 mm^3^) and 55% (average vol ~ 220 mm^3^) tumor reduction as compared to the control group (average vol ~ 480 mm^3^). These findings indicate that free therapeutic adenoviral vectors can suppress the tumor growth, but much efficient antitumor activity of adenoviral vectors can be achieve by mediating their delivery to tumors through MSCs-E1s (Fig. 8a, b).

### Apoptosis induction by replication-defective adenoviral vectors loaded MSCs-E1s in vivo

**Fig. 9:**
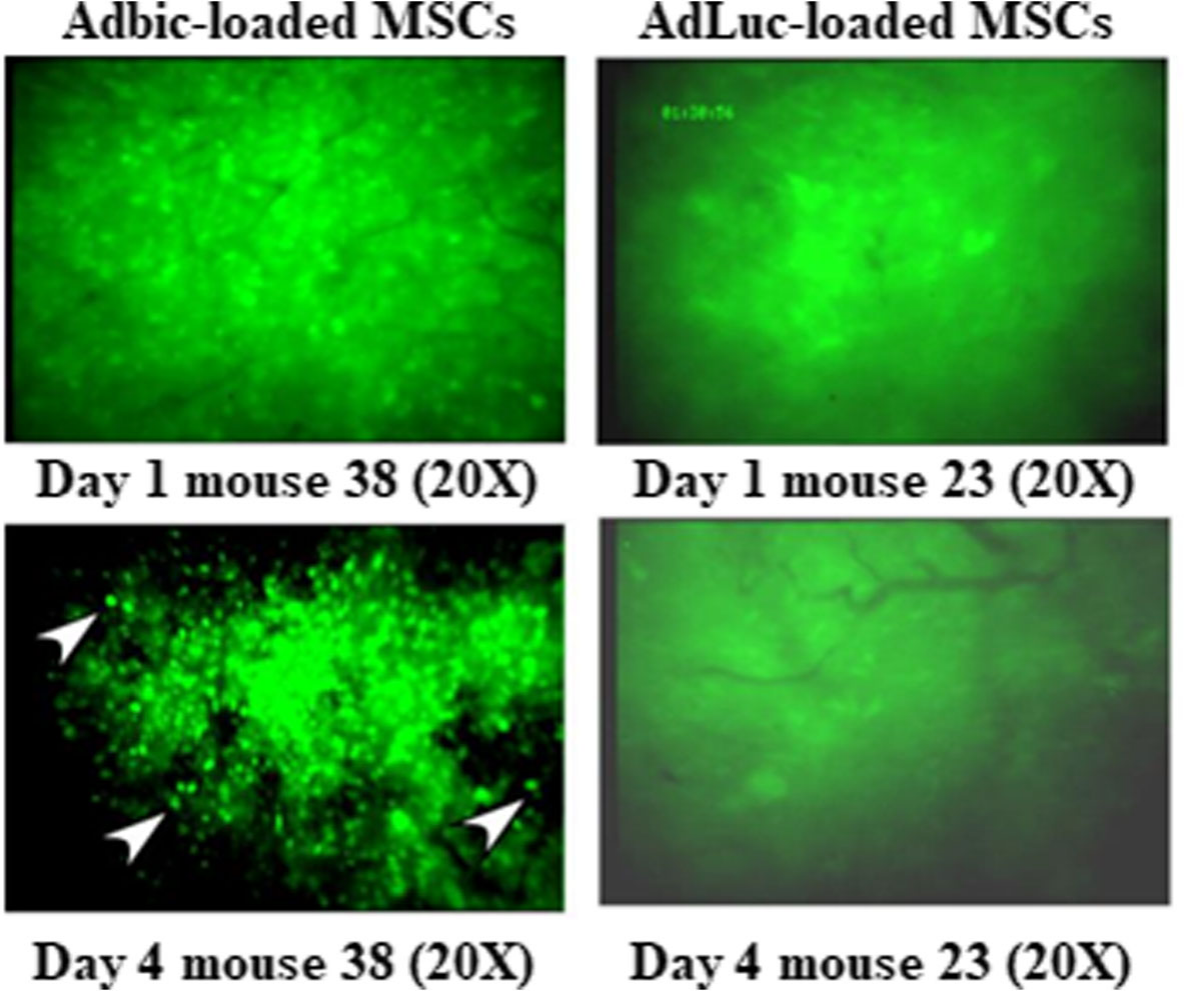
Apoptosis induction by replication-defective adenoviral vectors loaded MSCs-E1s in TRAMPC2 H2B/GFP prostate tumors models. MSCs-E1s (5 × 10^5^) loaded with 3 × 10^7^ pfu/cell of Adbic (left) or AdLuc (3 × 10^7^ pfu/cell) control (right) administered by a tail vein to male nude mice bearing TrampC2 tumors growing in dorsal skinfold chambers. GFP fluorescence was observed by intravital microscopy after MSCs-E1s injection (day 1) and on day 4. On day four, microscopic image of tumor in mice model that received Adbic-loaded MSCs-E1s showed typical bright, condensed nuclei (apoptosis) as indicated by the arrows. No such changes were observed in the control group that received Adluc-loaded MSCs-E1s

We investigated the apoptosis induction by MSCs-E1s loaded with Adbic in vivo*.* We injected 5 × 10^5^ MSCs-E1s loaded with Adbic 3 × 10^7^ pfu/cell to male nude mice group bearing TRAMPC2 prostate tumors growing in the dorsal skinfold chamber, and another TRAMPC2 prostate tumor model group was injected with 5 × 10^5^ MSCs-E1s loaded with Adluc 3 × 10^7^ pfu/cell (Control) via the tail vein. Nuclear changes indicative of apoptosis observed through intravital microscopy on days 1 and 4 of treatment in mice models given the treatment and compared with control. Microscopic images (× 20 magnification) of tumors on day 4 of treatment showed the typical bright, condensed nuclei (as indicated by arrow) thus confirming the induction of apoptosis while no such changes were observed in control mice models (Fig. 9). These findings suggest that MSCs-E1s successfully packaged, propagated, and delivered the adenoviral vectors to tumor cells and induced apoptosis.

## Discussion

Prostate cancer is the second leading cancer in men worldwide [1, 2]. Despite the availability of different treatment approaches, the development and application of targeted biological therapies with high efficacy and low adverse effects, especially for metastatic disease, are urgently needed. In this regard, adenoviral vector-based gene therapies (by transferring gene of interest to tumor sites, by exploiting the default oncolytic ability of adenoviruses), and packaging of these adenoviral vectors in vehicles having good tumor tropism, are effective and evolving approaches to address such issues [30–32].

Generally, in different adenoviral vector-based gene therapy, studies either conditionally replicating adenoviral vectors or replication-defective adenoviral vectors have been used. In this study, we have used both types of adenoviral vector-based gene therapy approaches. We devised a new therapeutic approach by combining adenoviral-based gene therapy with stem cell therapy. For such purpose, we used therapeutic adenoviral vector Adbic, which encodes two potent tumor suppressor genes p14ARF/p53. Externally aided combined enhanced expression of both tumor suppressor genes through Adbic achieved robust tumor suppression effects. The expression of p14ARF will stabilize the p53 protein and will let it perform its tumor suppression function efficiently. We also used the CRAd vector, which is an oncolytic adenoviral vector engineered for enhanced infectivity and tumor cell-specific replication. Gene therapy using both adenoviral vectors can offer the possibility of a competent, nontoxic therapeutic alternative for prostate cancer [25, 33]. To acquire maximum antitumor therapeutic effects of adenoviral vectors and to grapple the metastatic state of disease, we developed the novel MSC-based vehicle system which supports the packaging and propagation and offers the possibility of targeted delivery of adenoviral vectors to the tumor site. To ensure the propagation, we equipped the MSCs with adenovirus (Type C) E1A/E1B genes [34]. MSCs have been used for a long time as donor cells to repair damaged organs. It is currently evident that MSCs have the innate ability to migrate towards the primary and metastatic tumors and to home there. MSCs can also help the adenoviral vectors to evade the host immunoreactions thus increasing the availability of adenoviral vectors at tumor site [35, 36]. Possibility of genetic modifications and tumor tropism ability make MSCs a suitable choice for packaging, propagation, and delivering adenoviral vectors to tumor site.

We evaluated the antitumor activity of free CRAd or Adbic in vitro. Both the adenoviral vectors exhibited extraordinary antitumor activity against prostate tumor cells. Replication of CRAds is regulated by the survivin promoter, which enables selective replication of CRAds in the tumor cells. CRAds have shown effective antitumor activity against lung cancer, in vitro and in vivo [37]. Adenoviral vectors induced apoptosis in tumor cells. The elevated expression of p53 gene and its downstream genes that participate in the apoptosis pathway provided the concrete evidence that induction of apoptosis resulted due to enhanced p53 expression in tumor cells. P53 considered the main regulator of the cell cycle. Adenoviral vectors expressing p53 demonstrated antitumor activity against colorectal and breast tumors. Increased expression of p53 gene in adenoviral vector-treated tumor cells triggered the p53-associated apoptotic pathway [25, 38].

We subjected the mice prostate tumor models to only Adbic or Adbic in combination with chemotherapeutic agents to assess tumor suppressor activity. Adbic exhibited remarkable tumor suppressor activity by reducing the tumor growth in tumor models. P53 expressing adenoviral vectors can inhibit the growth of tumors by reducing the angiogenesis in tumors and inducing apoptosis in tumor cells [25, 31, 32].

We observed that adenoviral vectors chemo-sensitized the tumors and showed antitumor activity, but larger doses of vectors were required to achieve such results, and free adenoviral vectors showed poor intratumoral dissemination. Free adenoviral vectors might be sequestered by the reticuloendothelial system, resulting in less availability of adenoviral vectors at the tumor site [13, 14].

To address this issue and to fully exploit the antitumor effects of adenoviral vectors, we used MSCs. MSCs are easy to acquire, and these can selectively and precisely target the tumor cells due to their tumor tropism ability [18, 39–41]. MSCs can act as good carriers of adenoviral vectors.

We constructed MSCs-E1s by introducing the adenoviral E1A/E1B genes to MSCs. E1s support the replication of adenoviral vectors. MSCs-E1s presented good ability to package and propagate the adenoviral vectors, as high titers of progeny vectors recovered from infected MSCs-E1s. Tumor tropism of MSCs-E1s found satisfactory with no or very less normal tissue tropism of MSCs-E1s. MSCs are known to express receptors for several chemokines and growth factors secreted by tumor cells like platelet-derived growth factor (PDGF), interleukin 6 (IL-6), leucine-37 (LL37), prostaglandin E2 (PGE-2), and stromal cell-derived factor 1 (SDF-1). Interaction of these mediators with receptors on MSCs triggers the mobilization of MSCs towards tumors [42]. We also found meaningful homing and intratumoral dissemination of MSCs-E1s, revealed by intravital microscopy on TRAMPC2 H2B/GFP tumor models injecting with 3 × 10^6^ CMTMR-labeled MSCs-E1s.

Adenoviral vector-loaded MSCs-E1s exhibited better antitumor activity than adenoviral vector-loaded unmodified MSCs, in vitro. Since MSCs-E1s contained the E1A/E1B genes, which are essential for adenoviral replication. Presence of E1s in MSCs-E1s enabled high yield of progeny vectors than unmodified MSCs. Thus, a higher number of tumor cells were infected and killed.

In vivo analysis on mice prostate tumor models showed that both CRAds and Adbic-loaded MSCs-E1s successfully packaged, replicated, and delivered the adenoviral vectors to the tumor site. Vector-loaded MSCs-E1s integrated preferentially into the tumors and disseminated well within the tumors and suppressed the growth of prostate tumors in mice models.

## Conclusion

MSCs-E1s-mediated delivery of therapeutic adenoviral vectors may enable us to fully exploit the antitumor potential of these therapeutic adenoviral vectors for prostate cancer therapy. MSCs-E1s may circumvent the obstacles to systemically deliver therapeutic genes encountered with free adenoviral vectors, improve intratumoral dissemination of vector, and provide a highly effective, a nontoxic therapy for prostate cancer, and an effective alternative to conventional treatments for primary and metastatic disease.

## Abbreviations

Adbic: Replication-defective adenovirus expressing p14 and p53
AdGFP: Replication-defective adenovirus expressing green fluorescent protein
AdLuc: Adenovirus expressing Luciferase
BAX: Bcl-2-associated X protein
BCL2: B cell lymphoma 2
CMTMR: 5-(and-6)-(((4-Chloromethyl)benzoyl)amino)tetramethylrhodamine
CRAd: Conditionally replicating adenovirus
DMEM: Dulbecco’s modified Eagle’s medium
ELISA: Enzyme-linked immunosorbent assay
Mdm2: Mouse double minute 2 homolog
MOI: Multiplicity of infection
MSC: Mesenchymal stem cells
MSCs-E1s: E1A/E1B-modified mesenchymal stem cells
MTT: 3-(4,5-Dimethylthiazol-2-yl)-2,5-diphenyltetrazolium bromide
